# Leaves of the Arabidopsis maltose exporter1 Mutant Exhibit a Metabolic Profile with Features of Cold Acclimation in the Warm

**DOI:** 10.1371/journal.pone.0079412

**Published:** 2013-11-05

**Authors:** Sarah J. Purdy, John D. Bussell, Christopher P. Nunn, Steven M. Smith

**Affiliations:** 1 Australian Research Council Centre of Excellence in Plant Energy Biology, The University of Western Australia, Perth, Western Australia, Australia; 2 Institute of Biological, Environmental & Rural Sciences, Aberystwyth University, Aberystwyth, United Kingdom; University of Massachusetts Amherst, United States of America

## Abstract

**Background:**

Arabidopsis plants accumulate maltose from starch breakdown during cold acclimation. The Arabidopsis mutant, *maltose* *excess1-1*, accumulates large amounts of maltose in the plastid even in the warm, due to a deficient plastid envelope maltose transporter. We therefore investigated whether the elevated maltose level in *mex1-1* in the warm could result in changes in metabolism and physiology typical of WT plants grown in the cold.

**Principal Findings:**

Grown at 21 °C, *mex1-1* plants were much smaller, with fewer leaves, and elevated carbohydrates and amino acids compared to WT. However, after transfer to 4 °C the total soluble sugar pool and amino acid concentration was in equal abundance in both genotypes, although the most abundant sugar in *mex1-1* was still maltose whereas sucrose was in greatest abundance in WT. The chlorophyll *a*/*b* ratio in WT was much lower in the cold than in the warm, but in *mex1-1* it was low in both warm and cold. After prolonged growth at 4 °C, the shoot biomass, rosette diameter and number of leaves at bolting were similar in *mex1-1* and WT.

**Conclusions:**

The *mex1-1* mutation in warm-grown plants confers aspects of cold acclimation, including elevated levels of sugars and amino acids and low chlorophyll a/b ratio. This may in turn compromise growth of *mex1-1* in the warm relative to WT. We suggest that elevated maltose in the plastid could be responsible for key aspects of cold acclimation.

## Introduction

To survive in temperate climates, cold-hardy plants such as Arabidopsis make rapid genetic and metabolic changes in response to a reduction in temperature [1], [2]. Changes in membrane structure and the accumulation of cryoprotective solutes ensure that the plant is able to survive frost events [3], [4], [2]. Over a longer period of time whole-plant morphological changes, such as increased leaf biomass and reduced water content are observed [5], [6]. These short and long-term acclimatory processes ensure that Arabidopsis can grow and complete its life cycle whilst experiencing sub-optimal temperatures.

Several genes involved in starch metabolism have been observed to increase in expression in response to rapid cooling [7] [8] [9]. This response is followed by the breakdown of starch and concurrent accumulation of maltose [4], [10], [2]. Maltose has been identified as a potential cryoprotectant at low temperatures and is also the only metabolite to exhibit circadian rhythms under continuous light conditions at 4 °C [7], [11]. The importance of starch breakdown in conferring freezing tolerance has been demonstrated using mutants defective in chloroplastic β-amylase, *BAM3* (*BMY8*), which is essential for maltose production, and *GLUCAN WATER DIKINASE1* (*SEX1*), which mediates the phosphorylation of amylopectin. In both cases the mutants exhibited a freezing-sensitive phenotype, providing evidence that maltose accumulation is important for cold acclimation [7], [12]. 

The metabolite profile of a plant under cold conditions can therefore reveal details of its level of stress or tolerance. In addition to maltose, other soluble sugars including sucrose, glucose and raffinose change in abundance during cold acclimation, as do a plethora of amino and organic acids [13], [2], [14], [11]. For example, an increase in the glycine (Gly): serine (Ser) ratio indicates impairment of the oxidative photosynthetic carbon (C_2_) cycle [15]. Accumulation of amino acids or soluble sugars may suggest a reduction in resource utilization and therefore impairment in growth or biomass accumulation [16], [17]. Conversely, failure to accumulate particular amino acids (such as proline) in response to cold, results in cold sensitivity in Arabidopsis [18]. 

The *MALTOSE EXCESS1* (*MEX1*) gene encodes a maltose transporter that is localized to the chloroplast envelope and is required for maltose export into the cytosol [19]. A *mex1* null mutant accumulates very high levels of maltose in the leaves compared to wild type (WT) and has a starch-excess phenotype [19], [20]. However, these observations have only been reported for plants grown in warm conditions. We hypothesized that if elevated maltose plays a role in adaptation to cold, then *mex1-1* plants in the warm might exhibit some features of cold acclimation. To test this hypothesis and to provide insight into cold adaptations regulated by maltose, we investigated plant growth and the content of chlorophyll, carbohydrates and amino acids in *mex1-1* plants in the warm (21 °C) and after cold acclimation (4 °C). 

## Materials and Methods

### Plant material and growth conditions

*Arabidopsis thaliana mex1-1* mutant seeds were kindly donated by S. C. Zeeman (ETH Zurich). Wild type *Arabidopsis* L. ecotype Columbia (Col-0), and *mex1-1* mutant seeds were sown on 0.25 L pots of compost. Seeds were stratified for 3 days at 4 °C and transferred to growth chambers (150*μ*E cm^-2^, 21°C) in a 12 h light:12 h dark photoperiod. For metabolite and carbohydrate analyses, plants were grown on compost as above for 34 days before transfer to 4°C in a 12 h light:12 h dark photoperiod. For morphological and leaf pigmentation phenotyping, plants were germinated as above and then maintained at 21 °C or transferred to 4°C after 14 days. For phenotyping, plants were also maintained in their respective growth conditions until bolting.

### Metabolite Extraction and GC-MS and LC-MS Analysis

#### Sample extraction

Plants were grown at 21°C for 34 days before transfer to 4°C for 5 days. Whole rosettes were harvested 11 h into the 12 h light period on these days, and immediately frozen in liquid nitrogen. Approximately 60 mg fresh weight (FW) ground samples were weighed into pre-chilled 2 mL microfuge tubes. Ground samples were extracted in 500 µL of 100% methanol that contained 10 µg/ml each of the internal standards D-sorbitol-^13^C_6_ and L-valine-^13^C_5_,^15^N valine, and 5 µg/ml each of 2-aminoanthracene and pentafluorobenzoic acid. The sample mixtures were vortexed for 30 s and incubated at 70°C for 15 min. Milli-Q Water (500 µL) was added to the extracts, vortexed for a further 30 s and then centrifuged for 15 min at 13,000 rpm at room temperature (RT, 23°C) to pellet cellular debris. Aliquots (usually 10 µL) from the sample extracts were dried *in vacuo* for subsequent derivatisation and analysis by GC-MS or LC-MS. 

#### GC-MS analysis of sugars

GC-MS was done based on Roessner et al. (2001) [21]. The dried samples were re-dissolved in the equivalent volume of 30 mg mL^-1^ methoxyamine hydrochloride in pyridine and derivatised at 37°C for 120 min with mixing at 500 rpm. The samples were then treated for 30 min with 20 µL *N*,*O*-bis-(trimethylsilyl)trifluoroacetamide (BSTFA) and 2.5 µL retention time standard mixture [0.029% (v/v) *n*-dodecane, *n*-pentadecane, *n*-nonadecane, *n*-docosane, *n*-octacosane, *n*-dotriacontane, *n*-hexatriacontane dissolved in pyridine] with mixing at 500 rpm. The derivatised samples were allowed to rest for 60 minutes prior to injection. Samples (1 µL) were injected using a hot needle technique into a GC-MS system comprised of a Gerstel 2.5.2 autosampler, a 7890A Agilent gas chromatograph and a 5975C Agilent quadrupole MS (Agilent, Santa Clara, USA). The MS was adjusted according to the manufacturer’s recommendations using *tris*-(perfluorobutyl)-amine (CF43). The GC was performed on a 30 m VF-5MS column with 0.2 µm film thickness and a 10 m Integra guard column (Varian, Inc, Victoria, Australia). The injection temperature was set at 250°C, the MS transfer line at 280°C, the ion source adjusted to 250°C and the quadrupole at 150°C. Helium was used as the carrier gas at a flow rate of 0.8 mL min^-1^. The analysis of samples was performed under the following temperature program; start at injection 70°C, a hold for 1 min, followed by a 7°C min^-1^ oven temperature, ramp to 325°C and a final 6 min heating at 325°C.

Mass spectra were recorded at 2 scans s^-1^ with an *m/z* 50-600 scanning range. Both chromatograms and mass spectra were evaluated using the Chemstation program (Agilent, Santa Clara, USA). Mass spectra of eluting compounds were identified using the commercial mass spectra library NIST 05 (http://www.nist.gov), the public domain mass spectra library of Max-Planck-Institute for Plant Physiology, Golm, Germany (http://csbdb.mpimp-golm.mpg.de/csbdb/dbma/msri.html) and the in-house Metabolomics Australia mass spectral library. All matching mass spectra were quantified using by determination of the retention time and authentic sugar calibration standards.

#### LC-MS analysis of amino acids

LC-MS was done essentially as described in Boughton et al. (2011) [22], modified for use with Agilent 6490 Electrospray Ionisation-Triple Quadrupole MS and UPLC standards. Standards were prepared from two stock solutions: (1) Amino acids, containing a standard mix of 25 amino acids in water and 0.1% formic acid; (2) a 2.5 mM stock solution of sulfur-containing compounds comprising glutathione and s-adenosylhomocysteine in water with 10 mM TCEP and 1 mM ascorbic acid. These solutions were mixed and diluted using volumetric glassware with water containing 10 mM TCEP and 1 mM ascorbic acid, 0.1% formic acid to produce the following series of combined standards: 0.1, 0.5, 1, 5, 10, 20, 50, 100 and 150 µM.

For derivatisation, 10 µL aliquots of each standard or sample was added to 70 µL of borate buffer (200 mM, pH = 8.8 at 25°C) containing 10 mM TCEP, 1 mM ascorbic acid and 50 µM 2-aminobutyric acid. The resulting solution was vortexed, then 20 µL of AQC reagent (200 mM dissolved in 100% ACN) was added and immediately vortexed. The samples were heated with shaking at 55°C for 10 minutes then centrifuged and transferred to HPLC vials containing inserts. Derivatized samples were quantitatively analysed using an Agilent 1290 Infinity LC-system coupled to an Agilent 6490 Electrospray Ionisation-Triple Quadrupole MS. Injection volumes of 1 µL of samples or standards were used. Ions were monitored in the positive mode using a MRM method optimized for each analyte. The source, collision energies and fragmentor voltages were optimized for each analyte by infusing a derivatised standard with LC eluent. The following source conditions were used: sheath gas temperature 400°C, gas flow 11 L min^-1^, nebulizer pressure 45 psi and capillary voltage 3500 V. An Agilent Zorbax Eclipse RRHD-C18 Rapid Resolution HT 2.1 x 100 mm, 1.8 µm column was used with a flow rate of 800 µL min^-1^, maintained at 40°C, resulting in operating pressures below 1000 bar with a 10 min run time. "A gradient LC method was used with mobile phases comprised of (A) water/ 0.1% formic acid and (B) acetonitrile/ 0.1% formic acid. The proportion of B in the gradient and the times (min) were: 1 % B (0 - 0.5 min), 1-10% B (0.5 - 3.5 min), 10-20% B (3.5 - 6.0 min), 20-100% B (6.0 - 6.1 min), 100% B (6.1 - 7.0 min), 100-1% B (7.0 - 7.1 min), 1% B (7.1 - 10.0 min).

These conditions provided suitable chromatographic separation of modified amino acids and although co-elution was observed for some of the species, this could be overcome by the mass-selective capabilities of the mass spectrometer using MRM. Agilent MassHunter Quantitative Analysis Software, Version 6.0 was used to quantify levels of amino acids. 

### Chlorophyll measurements

Two fully expanded rosette leaves of *mex1-1* and WT were harvested from plants kept at 21°C for 4 weeks (when all plants were bolting) or after transfer to 4°C for ~131 days (when all plants were bolting). Five biological replicates were harvested per genotype, per treatment. Samples were weighed and then frozen in liquid nitrogen. Chlorophyll was quantified as described by 23 with minor modifications. Frozen plant material was ground in a ball mill to a fine powder. 2 ml 80% (v/v) acetone was added to the powdered plant material and left to extract the chlorophyll in the dark for 30 min. Samples were centrifuged at 15,000 x g for 15 min to pellet cellular debris. Supernatant was removed and measured using a spectrophotometer (U-2810, Hitachi) and UV Solutions software. Absorbance was measured at 663 nm and 646 nm to measure chlorophyll *a* and *b*, respectively. Total chlorophyll *a* and *b* was determined using the following formulae: (12.7 x A_663_ - 2.69 x A_646_) x Volume / Weight = Chl *a* mg/g FW and (22.9 x A_646_ - 4.86 x A_663_) x Volume / Weight = Chl *b* mg/g FW.

### Anthocyanin quantification

Samples were harvested, weighed and ground as for chlorophyll. Anthocyanins were extracted and quantified according to [24]: Ground samples were extracted in a solution containing 0.1 M hydrochloric acid and 18% (v/v) propanol in a heat block at 99 °C for 90 s. Samples were centrifuged for at 15 000 x g for 15 min to pellet cellular debris. The supernatant was removed and measured in a spectrophotometer (U-2810, Hitachi) with UV Solutions software. Absorbance was measured at 535 nm and 650 nm and normalized to fresh weight using the formula: 

(Abs 530 – Abs 630) / weight = anthocyanin units /g FW, where one unit of anthocyanin equals one absorbance unit in 1 ml of extraction solution.

### Statistical analyses

Statistical analyses were performed using GenStat 13th Edition (VSN International) and Microsoft Excel. For metabolites, a two way ANOVA and accompanying Tukey test was performed to identify significant differences between genotypes and treatments. For carbohydrates, leaf pigments and morphology, Student’s two-tailed T-tests assuming unequal variances were performed. Significance for both tests was set at 0.05.

## Results

### Amino acid profiling at 21 °C and after cold acclimation at 4 °C

**Figure 1:**
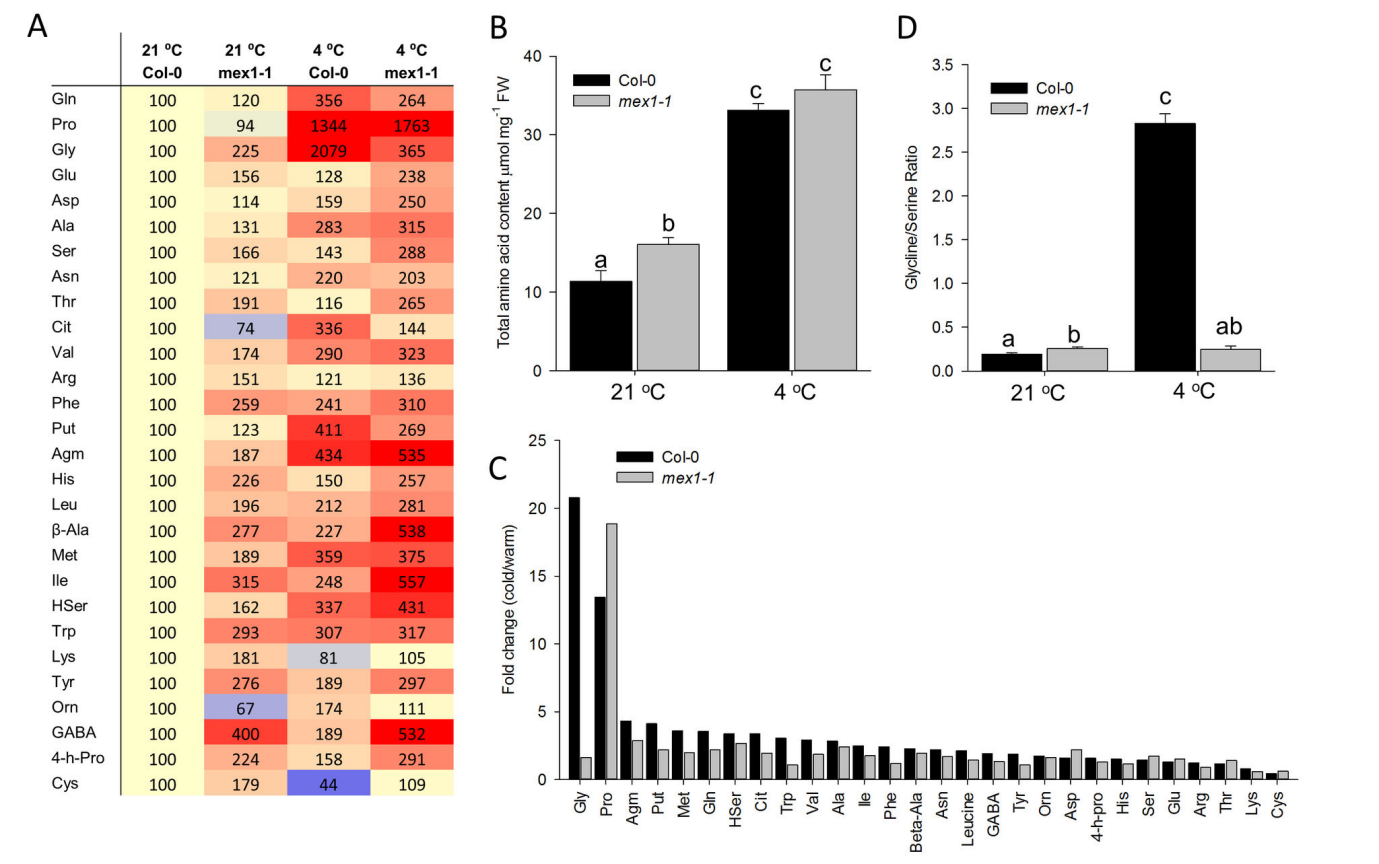
Changes in the amino acid profile of Col-0 and *mex1-1* rosette leaves grown at 21 °C and after cold acclimation at 4 °C for 5 days. A, Heatmap showing changes in amino acid abundance normalised to Col-0 in the warm. B, Total amino acid content before and after cold acclimation µmol mg^-1^ FW. C, Fold-change in amino acids (cold/warm). D, Glycine/Serine ratio before and after acclimation. D,. Data is the mean of 5 biological replicates, ± SE. Different letters indicate significant differences, Student’s T test P ≤ 0.05.

Nearly all amino acids showed an increase in abundance after cold acclimation (Table 1). The most abundant amino acid in WT was Gln both before and after acclimation whereas Glu and Pro were in greatest abundance in *mex1-1* in warm and cold conditions, respectively. In both treatments GABA was in lowest abundance in *mex1-1* and also in WT in the warm, after acclimation Ornithine was of lowest abundance in the WT (Table 1). To visualise the differences, and to compare amino acid levels in all treatments, we have also presented them in the form of a heatmap, normalised to the level of each amino acid in Col-0 in the warm (Figure 1A). Of the 28 amino acids quantified, 25 were present in greater amount in *mex1-1* than in WT in the warm, and of these, about half were present at levels equal to or greater than the levels in WT in the cold. The total amino acid abundance was 30%, higher in *mex1-1* than in WT in the warm but in the cold the total amino acid abundance increased to similar levels in both genotypes (Figure 1B). Thus, in amino acid content, *mex1-1* in the warm exhibits some characteristics of WT in the cold. Clear exceptions were Pro and its family members citrulline and ornithine. The amino acids most elevated in *mex1-1* in the warm do not fall into any particular chemical or biosynthetic classes, and their accumulation could in part be explained by slower growth of the mutant.

**Table 1:** Amino acid profile and abundance in Col-0 and mex1-1 grown at 21°C and after acclimation at 4°C for 5 days.

		**21 °C**		**4 °C**	
**amino acid:**	**abbreviation**	**col-0**	***mex1-1***	**col-0**	***mex1-1***
Glutamine	Gln	2525.3 ± 289.3^a^	3025.2 ± 221.7^a^	8992.5 ± 367.6^b^	6661.5 ± 624.6^c^
Proline	Pro	414.5 ± 60.6^a^	387.6 ± 165.4^a^	5571.3 ± 135.9^b^	7308.8 ± 442.2^c^
Glycine	Gly	249.2 ± 43.5^a^	560.2 ± 56.8^ab^	5181.4 ± 161.8^c^	909.5 ± 142.4^b^
Glutamic acid	Glu	2362.2 ± 287.8^a^	3677.0 ± 182.4^b^	3028.2 ± 106.6^ab^	5611.9 ± 395.2^c^
aspartic acid	Asp	1785.0 ± 309.4^a^	2040.9 ± 77.2^a^	2831.2 ± 114.8^b^	4464.1 ± 167.8^c^
Alanine	Ala	710.7 ± 132.0^a^	933.7 ± 108.1^a^	2012.4 ± 255.3^b^	2236.9 ± 328.6^b^
Serine	Ser	1287.2 ± 152.8^a^	2140.5 ± 89.1^b^	1841.1 ± 84.9^ab^	3706.4 ± 217.5^c^
asparagine	Asn	632.2 ± 77.6^a^	765.7 ± 60.8^a^	1390.5 ± 43.0^b^	1282.4 ± 106.5^b^
Threonine	Thr	849.6 ± 110.4^a^	1622.1 ± 129.8^b^	985.3 ± 25.7^a^	2248.9 ± 132.6^c^
Citrulline	Cit	101.2 ± 15.4^ac^	74.6 ± 4.6^a^	340.1 ± 18.0^b^	145.8 ± 9.3^c^
Valine	Val	73.4 ± 11.9^a^	128.0 ± 7.9^b^	212.6 ± 16.7^c^	237.3 ± 10.4^c^
Arginine	Arg	106.6 ± 29.3^a^	161.3 ± 13.6^a^	128.8 ± 10.1^a^	145.2 ± 17.0^a^
Phenylalanine	Phe	46.9 ± 6.3^a^	121.5 ± 8.0^bc^	113.0 ± 8.1^b^	145.6 ± 4.5^c^
Putrescine	Put	17.8 ± 3.6^a^	21.9 ± 1.8^a^	73.1 ± 5.3^b^	47.8 ± 5.5^c^
agmatine	Agm	13.7 ± 3.0^a^	25.6 ± 5.0^a^	59.4 ± 3.1^b^	73.3 ± 10.1^b^
Histidine	His	32.7 ± 4.2^a^	73.8 ± 13.9^b^	49.1 ± 9.8^ab^	84.2 ± 2.6^b^
Leucine	Leu	20.1 ± 2.6^a^	39.3 ± 1.6^b^	42.7 ± 3.3^bc^	56.4 ± 5.1^c^
β-alanine	β-Ala	18.5 ± 3.7^a^	51.3 ± 4.7^b^	42.0 ± 3.8^b^	99.5 ± 5.6^c^
Methionine	Met	11.0 ± 1.7^a^	20.8 ± 1.6^b^	39.5 ± 2.5^c^	41.2 ± 1.9^c^
Isoleucine	Ile	14.3 ± 6.0^a^	45.1 ± 4.0^ab^	35.5 ± 7.1^a^	79.6 ± 17.0^b^
Homoserine	HSer	9.1 ± 1.6^a^	14.7 ± 2.1^a^	30.7 ± 1.4^b^	39.2 ± 3.2^b^
Tryptophan	Trp	8.8 ± 1.1^a^	25.8 ± 1.9^b^	27.0 ± 1.5^b^	27.9 ± 2.4^b^
Lysine	Lys	18.5 ± 3.5^a^	33.4 ± 3.0^b^	15.0 ± 1.6^a^	19.5 ± 1.7^a^
Tyrosine	Tyr	6.3 ± 0.8^a^	17.4 ± 0.6^b^	11.9 ± 0.7^c^	18.7 ± 1.4^b^
Ornithine	Orn	4.6 ± 0.9^a^	3.1 ± 0.6^a^	8.0 ± 0.6^b^	5.1 ± 0.7^a^
gamma-aminobutyric acid	Gaba	3.8 ± 2.1^a^	15.2 ± 3.4^ab^	7.2 ± 2.5^ab^	20.2 ± 5.4^b^
4-hydroxy-proline	4-h-Pro	4.5 ± 0.8^a^	10.1 ± 0.8^bc^	7.1 ± 1.1^ab^	13.1 ± 1.7^c^
Cysteine	Cys	8.0 ± 1.6^a^	14.3 ± 0.6^b^	3.5 ± 0.5^c^	8.7 ± 1.1^a^

Quantities of each amino acid in rosette leaves are given as pmol mg-1 FW. Different letters indicate significant differences between genotypes and treatments (P= ≤ 0.05) as determined by Tukey’s Honestly Significant Difference test. Data is the mean of 5 biological replicates ± SE.

The greatest fold changes in abundance were for Gly in WT and Pro in both WT and *mex1-1* (Figure 1C). Although most of the amino acids were observed to increase, notable exceptions to this were Gly, which showed a 20-fold increase in the WT after acclimation but did not significantly increase in *mex1-1*, and Asp, which showed no change in abundance in the WT but increased in the mutant. In contrast, Lys and Cys exhibited slightly decreased abundance in both genotypes. 

The aspartate metabolic pathway includes synthesis of the amino acids Lys, Met, Thr, Ile and Gly [25]. The synthesis pathway is branched with one route leading to Lys and the other through homoserine (HSer) to Met, Thr and, via Thr, to Ile and Gly [25], [11]. There was no increase in Lys abundance in either genotype after transfer to 4 °C, whereas increases in HSer, Met, Ile, Thr were observed in both WT and *mex1-1* after cold treatment. Therefore, only amino acids in the aspartate pathway leading away from Lys biosynthesis were increased in response to cold. This is in agreement with previous reports [11]. The increase of Gly in WT in response to cold is indicative of impairment in the oxidative photosynthetic carbon (C_2_) cycle in which glycine, produced in peroxisomes, is converted within the mitochondria to Ser [26]. In WT grown at 21 °C, Gly was 20% of Ser abundance, whereas after cold acclimation Gly exceeded Ser by 3-fold (Figure 1D). In contrast to WT, a 2-fold increase in Gly was observed in *mex1-1* but serine also increased 2-fold in response to cold treatment, therefore the Gly:Ser ratio did not change in *mex1-1* after acclimation (Figures 1C & D). 

### Carbohydrate Profiling at 21 °C and after Cold Acclimation at 4 °C

**Figure 2:**
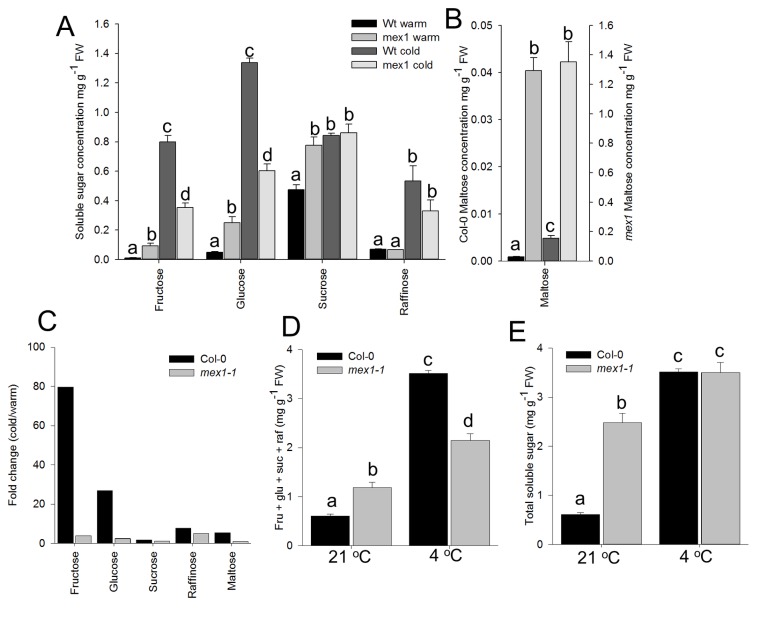
Soluble sugar profile of Col-0 and *mex1-1* rosette leaves grown at 21 °C and after cold acclimation at 4 °C for 5 days. A, Fructose, glucose, sucrose and raffinose concentration, B Maltose concentration. Note that the vertical axis is represented on different scales for wild type and *mex1-1*. C. Fold change of soluble sugars after transfer (cold/warm). D, sum of sugars metabolisable by both genotypes (fructose + glucose + sucrose + raffinose) and E, total soluble sugars (fructose + glucose + sucrose + raffinose + maltose). Concentrations are given as mg g^-1^ FW and values represent the mean ± SE of five biological replicates. Letters indicate significant differences between genotypes and treatments for each sugar or sugar pool (Student’s T-Test, P ≤ 0.05).

Under warm conditions *mex1-1* contained significantly more fructose, glucose, sucrose and maltose than WT (Figure 2A & B). Previous studies have reported that *mex1-1* had a maltose level more than 40-fold that of WT. Our values were substantially more than that, mainly owing to the lower values for WT plants than previously reported [19]. Our finding that *mex1-1* contained about 1.2 mg g^-1^ FW in warm conditions is in agreement with previous reports of 1-2 mg g^-1^ FW [19], [20] . 

After transfer to cold conditions soluble sugars increased in both genotypes but fructose, glucose, sucrose and maltose increased to a greater extent in the WT than in *mex1-1* (Figures 2A, B & C). Although a change in abundance of raffinose was observed, concentrations remained similar between the two genotypes (Figure 2A). After acclimation the most abundant soluble sugar in WT was glucose, whereas in *mex1-1* maltose was still in greatest abundance, followed by sucrose (Figure 2A).

In warm conditions *mex1-1* contained a greater total amount of sugars that could be metabolized by both genotypes (fructose, glucose, sucrose and raffinose), but after cold acclimation Col-0 contained approximately 40% more of these carbohydrates than *mex1-1* (Figure 2D). Under warm conditions the sum of all metabolisable sugars + maltose was, unsurprisingly, greater in *mex1-1* by approximately 5-fold, but intriguingly, after cold acclimation the total sum of carbohydrates was similar between the two genotypes (Figure 2E). 

### Leaf pigmentation

**Figure 3:**
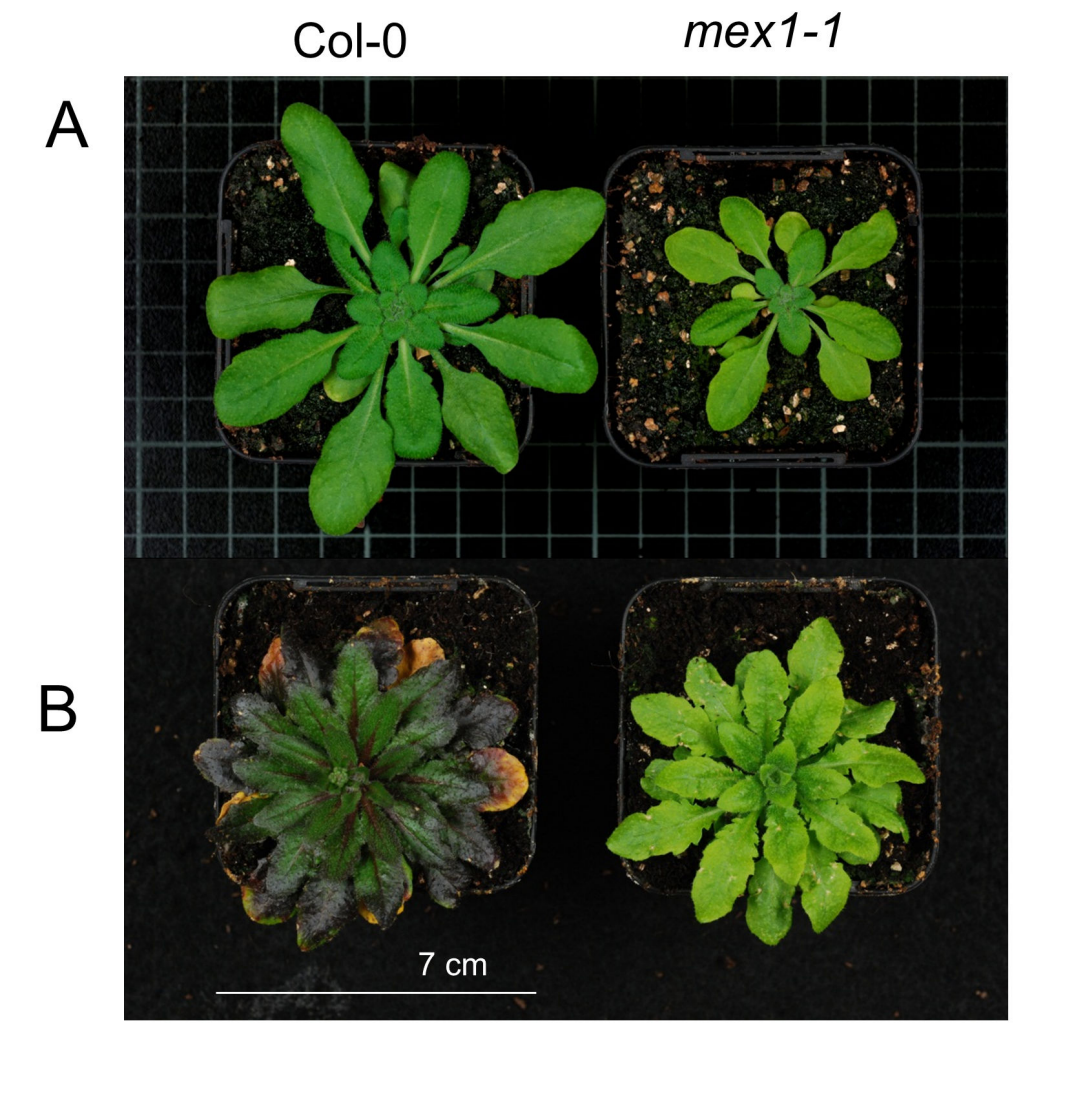
Physical appearance of Col-0 and *mex1-1* mutants when grown for 39 days at 21 °C (A) or for ~131 days until bolting at 4 °C (B).

After growth until bolting at 21 °C, *mex1-1* plants were smaller and less green in appearance than the WT (Figure 3A), consistent with previous reports [19], [20]. At the onset of a long-term cold treatment the *mex1-1* plants were substantially smaller than WT. However, after transfer to 4 °C and growth for several weeks until bolting, *mex1-1* plants were still paler than WT, but they grew to be of comparable size. Unlike wild type, cold-grown *mex1-1* showed very little leaf senescence or anthocyanin accumulation (Figure 3B). 

**Figure 4:**
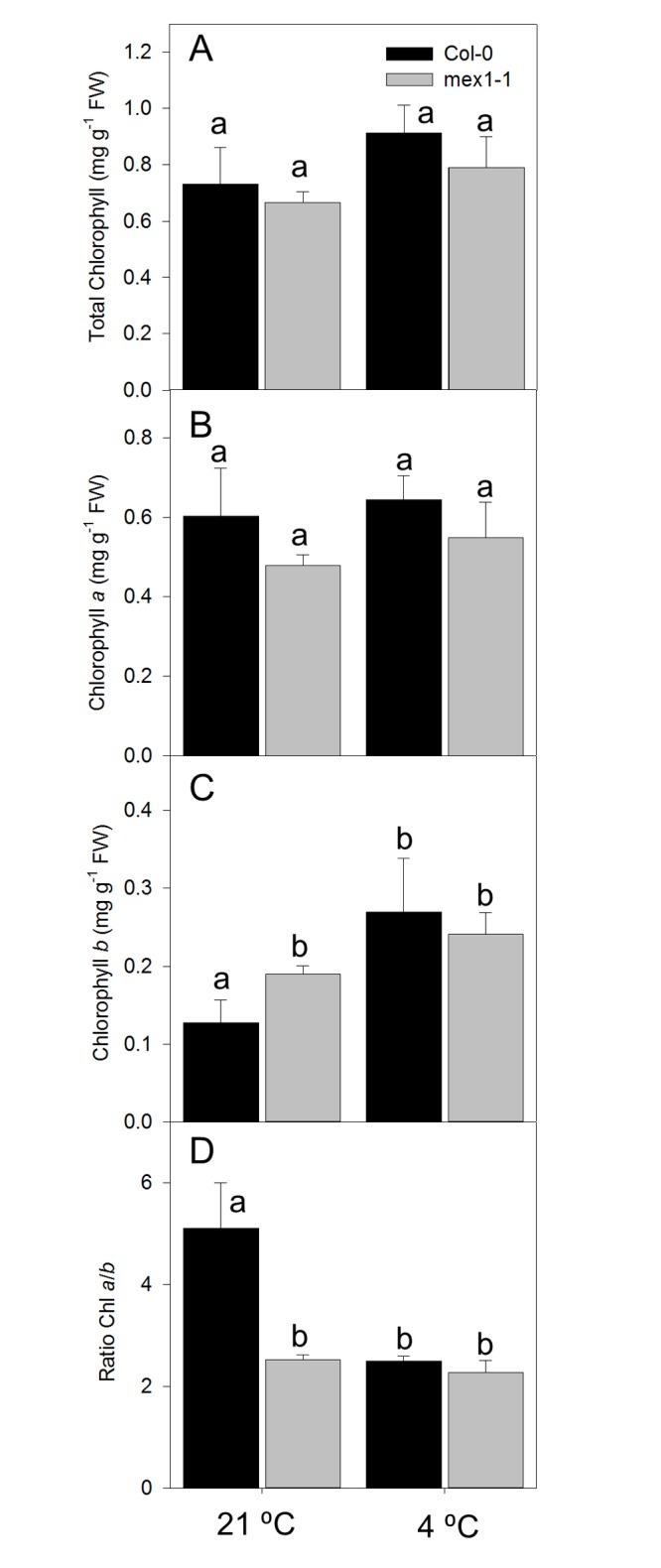
Chlorophyll content of WT and *mex1-1* plants. (A) Total chlorophyll, (B) chlorophyll *a* content, (C) chlorophyll b content, and (D) chlorophyll *a*/*b* ratio of plants grown at 21°C and 4°C. Values represent the mean ± SE of five biological replicates Different letters show significant differences (Student’s T-Test P ≤ 0.05).

The quantification of chlorophyll from the mutant and WT showed some remarkable features. At either 21 °C or 4 °C the total concentration of chlorophyll (mg/g^-1^ FW) in the WT was not significantly different to that of *mex1-1* (Figure 4A). Therefore the pale appearance of *mex1-1* may be explained by thinner leaves, or lack of other pigments. Consistent with this *mex1-1* reportedly has approximately 25% less chlorophyll than wild type on a leaf area basis, and leaves that were only 77% as thick as those of the WT [20]. Irrespective of growth temperature, there was no significant difference in the abundance of Chl *a* in either genotype (Figure 4B). However, there was a greater abundance of Chl *b* in *mex1-1* compared to WT at 21 °C (Figure 4C) which resulted in a reduction in the Chl *a*/*b* ratio in *mex1-1* to about half that of WT (Figure 4D). In contrast, the Chl *a/b* ratios at 4 °C were the same in *mex1-1* and WT (Figure 4D). This was due to a 2-fold increase in Chl *b* abundance in the WT at 4 °C. Therefore, a feature of WT plants grown in the cold is a lower Chl *a/b* ratio than when maintained at 21°C. In contrast *mex1-1* has a low Chl *a/b* ratio at 21°C and in this respect is characteristic of a WT plant growing at 4°C. 

**Figure 5:**
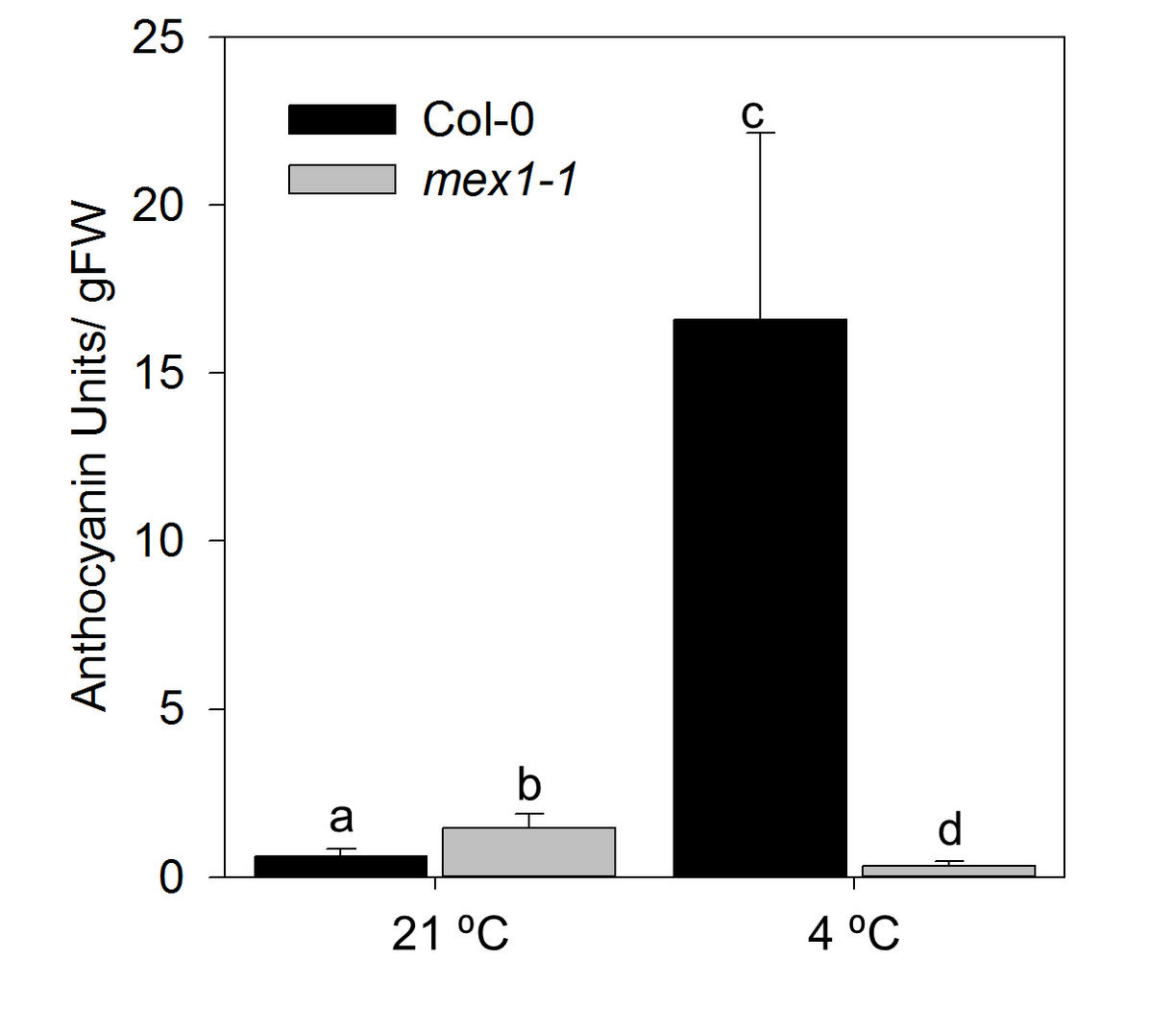
Total anthocyanin content in Col-0 and *mex1-1* when grown to bolting at 21 °C or 4 °C. Data is the mean of five biological replicates ± SE. Different letters show significant differences (Student’s T-Test P ≤ 0.05).

The anthocyanin content of the WT was 26-fold higher after growth at 4 °C while the content in *mex1-1* was low in both treatments (Figure 5). Accumulation of anthocyanins in response to cold is a common response in plants and presumably provides an adaptive advantage [27]. Thus *mex1-1* would appear to be deficient in this aspect of the response to cold. 

### Shoot growth and Morphology

**Figure 6:**
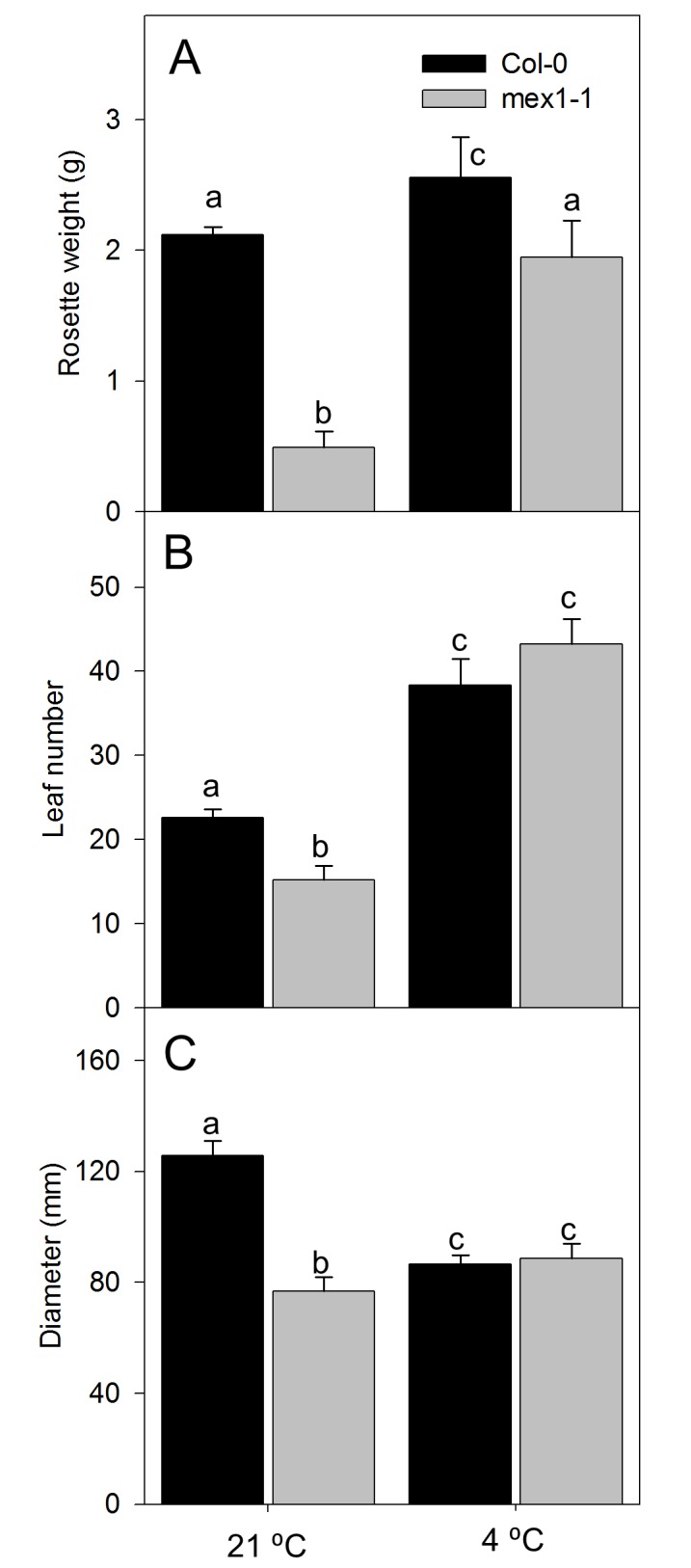
Morphology of Col-0 and *mex1-1* grown to bolting at 21 °C and 4 °C. (A) Rosette weight (g per plant), (B) Average leaf number at bolting and (C) Rosette diameter (mm). Data is the mean of at least 9 biological replicates ± SE. Different letters show significant differences (Student’s T-Test P ≤ 0.05).

Quantitative measurements of rosette mass at the time of bolting showed that at 21 °C *mex1-1* was only 25% the mass of wild type (Figure 6A). This is explained in part by fewer, smaller leaves (Figure 6B, C). In contrast, after growth to bolting at 4 °C *mex1-1* rosettes had 4-fold greater biomass compared to those grown at 21 °C, whereas cold-grown WT plants had only slightly (1.2-fold) more biomass at bolting than warm grown WT plants. Indeed, in the cold, *mex1-1* was 80% of the biomass of WT plants at bolting (Figure 6A). At 4 °C, there were nearly three times as many leaves on *mex1-1* plants than at 21°C, but less than 2-fold more on wild type plants (Figure 6B). Rosette diameter increased in the cold in *mex1-1* to be similar to WT (Figure 6C). These morphological changes meant that, in contrast to 21 °C, no differences in leaf number and rosette diameter were observed between *mex1-1* and WT at 4 °C. Therefore the rosette morphology of cold-treated WT and *mex1-1* plants is similar when grown at 4 °C. 

## Discussion

The mutant *mex1-1* has elevated maltose in the plastid under optimal growing conditions [19], [20]. We used this mutant to test the hypothesis that elevated maltose could induce changes characteristic of adaptation to cold. Although growth of *mex1-1* at 21°C was quite reduced compared to Col-0, we found that *mex1-1* plants appeared physically less compromised than WT when grown at 4 °C. At 21 °C, *mex1-1* had elevated concentrations of total amino acids, glucose, fructose, sucrose and maltose compared to WT. All of these metabolites increased markedly in the WT in response to chilling, as previously reported [28], [11], [2]. Their response in *mex1-1* was much less pronounced, and indeed sucrose levels did not increase in *mex1-1* during acclimation. Therefore under warm conditions, *mex1-1* exhibited a metabolic phenotype with similarity to a cold-acclimated WT. 

When the genotypes were transferred to 4 °C at the onset of the experiment, *mex1-1* was markedly smaller than WT but by the onset of bolting in the cold, *mex1-1* was 80% the mass of WT. This suggests that at 4 °C the growth rate of *mex1-1* was similar to WT. Several attributes of the metabolomes of each genotype are indicative of a shift in growth rate, and by extension, final biomass. Firstly, a higher abundance of amino acids has been correlated with reduced biomass in Arabidopsis [17], this is in agreement with the observation that the *mex1-1* plants grown in the warm had elevated levels of total amino acids and a slower growth rate [19]. However, after cold acclimation the abundance of total amino acids was similar in the two genotypes and plant sizes were more equal, suggesting more similar growth rates. Secondly, the abundance of Chl *b*, but not Chl *a*, has also been negatively correlated with biomass which is consistent with the dwarf phenotype of *mex1-1* in the warm and the reduced rosette size of both genotypes in the cold [17]. Finally, a negative correlation between the abundance of the amino acid Gln and growth rate has been reported in Arabidopsis [16]. Under warm conditions Gln was elevated in *mex1-1* compared to WT, consistent with a slower growth rate. However, after cold acclimation Gln increased 2-fold in the mutant and 4-fold in WT, so levels became higher in the WT. An increase in the growth rate of acclimated *mex1-1* compared to WT implies a reduction in the impairments inflicted by the mutation when the mutant is grown in the cold.

Our results point to a re-programming of carbon metabolism within *mex1-1* that is similar to that observed in cold acclimated WT plants. During the process of cold acclimation, leaves that have developed in the warm experience changes in carbon metabolism that involve an increase in activity of enzymes involved in the photosynthetic carbon reduction cycle, such as cytosolic Fru-1,6-BPase, and sucrose synthesis, such as sucrose phosphate synthase (SPS) [29]. Increases in the activity of enzymes involved in starch synthesis, such as ADP-Glc-pyrophosphorylase (AGPase) have also been observed, but not to the extent of those involved in the cytosolic carbon metabolic pathway [29]. These results suggest that cold acclimation involves a transition away from plastidial, starch-derived carbon sources and towards soluble cytosolic carbon reserves [29]. In our study we observed elevated levels of metabolisable sugars (glucose, fructose, sucrose) within *mex1-1* leaves under warm conditions. This finding complements previous reports in which elevated turnover of soluble sugars and reduced turn-over of starch through the diurnal cycle were observed in *mex1-1* in warm conditions [19], [30]. Furthermore, the level of sucrose in warm grown *mex1-1* plants was equal to that in cold acclimated WT. This suggests that, owing to the impairment in maltose export, leaves of *mex1-1* have already shifted from a reliance on plastid-starch derived carbon to a greater utilization of cytosolic carbon sources, similar to the adaptations made in a cold acclimating WT plant [29]. 

Our observations of *mex1-1* are similar to those reported for the Arabidopsis *eskimo1* mutant that, unlike WT plants, exhibits constitutive freezing tolerance without requiring a period of acclimation [31]. Under warm growing conditions the *eskimo1* mutant was dwarf, grew more slowly and had elevated levels of Pro and soluble sugars compared to WT [31], [32]. However, after 60 days at 4 °C, *eskimo1* was similar in size and had grown at the same rate as WT [31]. In these respects the phenotype of *eskimo1* bears similarity to *mex1-1*. It would be interesting to investigate the diurnal patterns of soluble sugar and starch metabolism in *eskimo1* to see whether it has a greater reliance on cytosolic derived carbon rather than plastidial starch. Similarly, it would be worth testing the freezing tolerance capacity of *mex1-1* to further explore these similarities.

Glycine has been observed to increase in WT plants in response to chilling in a number of studies [2], [11]. Therefore, the failure of *mex1-1* to accumulate high levels of Gly in response to cold acclimation suggests that *mex1-1* is deficient in this aspect of cold acclimation. As cold acclimation is part of the adaptive process that prepares the plant to withstand freezing temperatures, it is feasible that this deficiency may confer greater susceptibility to freezing stress. As a freezing sensitive phenotype has been reported for the starch metabolism mutants β*–amylase3*/*bmy9* and *sex1* it would be interesting to determine whether they are also deficient in the accumulation of Gly during cold acclimation [10], [8].

Plants possess signalling mechanisms that allow them to regulate the accumulation of nutrients according their metabolic status. For example, when carbohydrates are in abundance photosynthesis is down-regulated, or if internal nitrogen levels are sensed to be low, nitrate uptake and reduction is stimulated [33]. Cross-talk exists between these two signalling pathways to maintain a balance between these two essential nutrients [33]. As carbohydrate levels are high in *mex1-1*, it is feasible that this stimulates greater uptake and reduction of nitrates resulting in the increased abundance of N storing amino acids, such as Gln and Glu, as observed under warm conditions. However, as *mex1-1* is dwarf under warm conditions it is clearly unable to capitalize on its high nutrient status for growth, possibly owing to carbon-limitation at night [20]. 

The pale green appearance of *mex1-1* has been explained as due to reduction in the number of chloroplasts, thinner leaves and chloroplast degradation [20]. In the present study we also found that *mex1-1* had a lower chlorophyll *a/b* ratio than the wild type when grown in warm conditions. However, when grown in the cold, the chl *a/b* ratio of the WT was reduced significantly, but that of *mex1-1* remained unaltered. It has been reported that the chlorophyll *a/b* ratio of tomato plants exposed to elevated CO_2_ for 9 days was reduced whilst the content of soluble sugars and starch increased [34]. These results suggest that reduction in the chlorophyll *a/b* ratio may occur as a result of elevated soluble sugars, as observed in *mex1-1* in the warm and both *mex1-1* and WT in the cold. 

The lack of accumulation of anthocyanins in *mex1-1*, particularly after exposure to cold, is surprising as it has previously been reported that treating Arabidopsis seedlings with sucrose or maltose resulted in greatest accumulation of anthocyanins compared to other carbohydrates [35] [36], [37]. Furthermore, the up-regulation of genes involved in flavonoid biosynthesis has been reported in *mex1-1*, but consistent with our findings no concomitant increase in anthocyanin accumulation was observed [20]. Exactly why *mex1-1* does not accumulate anthocyanins is unclear; one possibility is that the site of maltose accumulation, e.g in the plastid or cytosol, may be important to stimulate anthocyanin accumulation. The cytosolic glucosyltransferase mutant, *dpe2*, accumulates elevated levels of maltose in both the plastid and cytosol and has reduced chlorophyll content per unit area [38], [20]. It would therefore be revealing to test whether this mutant retains the ability to synthesize anthocyanins in the cold. A second possibility is that the chloroplast degenerative processes that occur in *mex1-1*, and give rise to its chlorotic appearance [20], may also serve to degrade anthocyanins, or their precursors. 

In summary we have discovered that despite the differences between wild type and *mex1-1* mutant plants grown at 21°C, the mutant shows greater similarity to WT, in terms of biomass, amino acids, total carbohydrates and chlorophyll when grown in the cold. This is probably the result in *mex1-1* of a pre-existing shift away from plastidial starch-derived carbon to cytosolic carbon reserves that mirrors changes occurring in plants undergoing cold acclimation. The results further suggest that maltose accumulation could, in part, mediate some of the effects of chilling on plant growth, including changes to chlorophyll *a/b* ratio and amounts of amino acids and sugars. 
